# Comparison of Predictive Properties between Tools of Patient-Reported Outcomes: Risk Prediction for Three Future Events in Subjects with COPD

**DOI:** 10.3390/diagnostics13132269

**Published:** 2023-07-04

**Authors:** Koichi Nishimura, Masaaki Kusunose, Ryo Sanda, Mio Mori, Ayumi Shibayama, Kazuhito Nakayasu

**Affiliations:** 1 Visiting Researcher, National Center for Geriatrics and Gerontology, 7-430 Morioka-cho, Obu 474-8511, Japan; 2 Clinic Nishimura, 4-3 Kohigashi, Kuri-cho, Ayabe 623-0222, Japan; 3 Department of Respiratory Medicine, National Center for Geriatrics and Gerontology, 7-430 Morioka-cho, Obu 474-8511, Japan; kusunose@ncgg.go.jp (M.K.); ryo-sand@ncgg.go.jp (R.S.); mio-mori@ncgg.go.jp (M.M.); 4 Department of Nursing, National Center for Geriatrics and Gerontology, 7-430 Morioka-cho, Obu 474-8511, Japan; ayuminarita3@gmail.com; 5 Data Research Section, Kondo P.P. Inc., 17-25, Shimizudani-cho, Tennoujiku, Osaka 543-0011, Japan; nakayasu@mydo-kond.co.jp

**Keywords:** COPD, dyspnea, health status, surveys and questionnaires, mortality, quality of life

## Abstract

Background: Patient-reported outcome (PRO) measures must be evaluated for their discriminatory, evaluative, and predictive properties. However, the predictive capability remains unclear. We aimed to examine the predictive properties of several PRO measures of all-cause mortality, acute exacerbation of chronic obstructive pulmonary disease (COPD), and associated hospitalization. Methods: A total of 122 outpatients with stable COPD were prospectively recruited and completed six self-administered paper questionnaires: the COPD Assessment Test (CAT), St. George’s Respiratory Questionnaire (SGRQ), Baseline Dyspnea Index (BDI), Dyspnoea-12, Evaluating Respiratory Symptoms in COPD and Hyland Scale at baseline. Cox proportional hazards analyses were conducted to examine the relationships with future outcomes. Results: A total of 66 patients experienced exacerbation, 41 were hospitalized, and 18 died. BDI, SGRQ Total and Activity, and CAT and Hyland Scale scores were significantly related to mortality (hazard ratio = 0.777, 1.027, 1.027, 1.077, and 0.951, respectively). The Hyland Scale score had the best predictive ability for PRO measures, but the C index did not reach the level of the most commonly used FEV_1_. Almost all clinical, physiological, and PRO measurements obtained at baseline were significant predictors of the first exacerbation and the first hospitalization due to it, with a few exceptions. Conclusions: Measurement of health status and the global scale of quality of life as well as some tools to assess breathlessness, were significant predictors of all-cause mortality, but their predictive capacity did not reach that of FEV_1_. In contrast, almost all baseline measurements were unexpectedly related to exacerbation and associated hospitalization.

## 1. Introduction

The importance of patient-reported outcome (PRO) measures when evaluating healthcare delivery and conducting scientific investigations has grown substantially [1,2,3,4]. Chronic obstructive pulmonary disease (COPD) is considered a model for the evaluation of PRO measures [5,6]. Guyatt and colleagues first developed the Chronic Respiratory Disease Questionnaire (CRQ) in 1987 to measure the disease-specific quality of life of individuals with COPD [7]. The updated Global Initiative for Chronic Obstructive Lung Disease (GOLD, 2011) proposed that symptoms be evaluated as health status measures using the COPD Assessment Test (CAT) [8,9,10], one of the PRO measuring tools recommended in clinical practice according to the international document [11]. PRO measures are thus considered essential when assessing a patient’s COPD.

Health indicators, including PROs, can be discussed from three perspectives. First, they can differentiate between lesser and more severely ill patients (discriminatory quality). Second, they can measure the amount of change (an evaluative feature). Third, they can forecast future outcomes (predictive property). In the twentieth century, forced expiratory volume in one second (FEV_1_) and age were believed to be the strongest mortality predictors in subjects with COPD [12]. Subsequently, several predictors of mortality have emerged in the literature, including dyspnea, health status, exercise capacity, and physical activity [13,14,15,16]. Oga and colleagues reported that the St. George’s Respiratory Questionnaire (SGRQ) Total score was able to predict mortality for a period of up to five years, but CRQ scores were not associated with seven-year mortality [15,17]. As COPD is a progressive disease, it is important to consider future outcome predictors such as FEV_1_ and PRO measures when assessing disease severity, as these can be used to predict mortality.

We hypothesized that individual PRO measures had been independently based on specific conceptual frameworks and are not interchangeable, although several PRO tools aimed at subjects with COPD have been reported in the literature [4,18]. For example, the SGRQ and CAT were developed to measure COPD-specific health status [8,9,10,19], Evaluating Respiratory Symptoms in COPD (E-RS) focuses on respiratory symptoms [20,21], and Dyspnoea-12 (D-12) is targeted at breathlessness [22,23,24,25]. They have generally been administered to subjects with COPD individually or in combination when multifaceted analysis and evaluation of outcome markers is required. It remains unclear whether the currently used PRO measures can predict future outcomes for patients with COPD and whether or not the predictive properties are different from the perspective of individual conceptual frameworks. The purpose of this study was to examine the predictive properties of several different PRO measures in subjects with COPD and to investigate which of these measures best predicts mortality, acute exacerbation of COPD (AECOPD), and hospitalization due to AECOPD.

## 2. Methods

### 2.1. Participants

A total of 122 stable COPD patients were recruited from our outpatient clinic at the Department of Respiratory Medicine of the National Center for Geriatrics and Gerontology between April 2013 and April 2019 and followed up to December 2019 for a maximum of six and a half years. The criteria for inclusion were over 50 years old, former or current smokers with a cumulative smoking history of more than 10 pack-years, and chronic fixed airflow limitation (described elsewhere as a part of the hospital-based cohort study) [26]. The exclusion criteria included an exacerbation of COPD in the preceding three months.

### 2.2. Measurements

All eligible subjects completed lung function tests and PRO measurements at baseline on the same day. Participants were instructed to arrive at the study site at least 12 h after stopping bronchodilator use. During the visit, they were monitored by a physician while they inhaled long-acting bronchodilators and, more than 60 min later, underwent spirometry with the CHESTAC-8800 spirometer (Chest, Tokyo, Japan). The test was performed in the sitting position, and the highest values of the three measurements were analyzed. Residual volume was calculated using the closed-circuit helium method, and diffusing capacity for carbon monoxide (DL_CO_) was determined using the single-breath technique [27].

The survival of all registered subjects was assessed until mid-December 2019. For those who did not attend an outpatient clinic, telephone or postal contacts with families or primary health practitioners were used to obtain information on mortality. Those whom we could not reach were regarded as having withdrawn. The period from entry to the last participation or event was recorded for analysis. AECOPD was defined as a worsening of respiratory symptoms requiring treatment with systemic corticosteroids or antibiotics, or both [28].

### 2.3. Patient-Reported Measurements

Disease-specific health status was assessed with the CAT and SGRQ [8,9,10,19]. The CAT scores range from 0 to 40, with a score of zero indicating no impairment [8,9,10]. The SGRQ consists of 50 items, divided into three components: Symptoms, Activity and Impact, and the Total score is calculated [19]. Higher scores on the SGRQ indicate a more severely impaired health status. To assess the severity of breathlessness, we used the Baseline Dyspnea Index (BDI) and the Dyspnoea-12 (D-12) [22,23,24,29]. The BDI is composed of three categories: functional impairment, magnitude of task and magnitude of effort, rated by five grades from 0 (severe) to 4 (not impaired) for each [29]. The D-12 consists of 12 elements (7 physical and 5 emotional), and the D-12 Total scores range from 0 to 36, with higher scores denoting more severe dyspnea [22,23,24]. The total score of the E-RS indicates the severity of respiratory symptoms in general, where scores range from 0 to 40, with higher scores indicating more severe symptoms [20,21]. Three subscales are used to assess breathlessness (RS-Breathlessness), cough and sputum (RS-Cough and Sputum), and chest-related symptoms (RS-Chest Symptoms) [20,21]. Although E-RS is intended to be administered using accredited electronic devices, none were available in the Japanese version. Global health was also assessed using the Hyland Scale with scores ranging from 0 to 100, where 0 = ‘might as well be dead’ and 100 = ‘perfect quality of life’ [30]. All the validated Japanese versions were self-administered using a paper-based questionnaire under site supervision in the aforementioned order (in booklet form).

### 2.4. Statistical Methods

All results are expressed as mean ± standard deviation (SD). A *p*-value of less than 0.05 was considered statistically significant. Differences between groups were determined by the Steel–Dwass and Kruskal–Wallis tests. Univariate Cox proportional hazards analyses were performed to investigate the relationships between measurements at baseline and subsequent events. Results of regression analyses are presented in terms of hazard ratio (HR) with the corresponding 95% confidence intervals (CI). They were first calculated by actual measured value and further analyzed in a standardized format using a score to show HR for changes per SD. The C-index of an event prediction model is the property of correctly discriminating between event and non-event-onset individuals and is often used when comparing different measures, e.g., when comparing different models. The closer the value of the C index is to 1.0, the better the risk prediction.

## 3. Results

### 3.1. Subject Characteristics and Scores Obtained at Baseline

During the study period, 122 consecutive patients (113 men) with mild to very severe COPD, with a wide range of FEV_1_ values, were investigated. The mean age and FEV_1_ were 74.5 ± 6.4 years and 1.72 ± 0.54 L (68.8 ± 20.3% pred), respectively. Ninety-four were former smokers, and 28 were current smokers. Patient characteristics and the results of the pulmonary function tests at baseline are shown in Table 1. According to the classification of GOLD airflow limitation [11], 41 subjects (33.6%) were included in GOLD 1 (defined as FEV_1_ ≥ 80% predicted), 60 (49.2%) in GOLD 2 (50% ≤ FEV_1_ < 80% predicted), 14 (11.5%) in GOLD 3 (30% ≤ FEV_1_ < 50% predicted) and 7 (5.7%) in GOLD 4 (FEV_1_ < 30% predicted) (Table 2). The elderly population was more strongly represented than expected, and there were only a small number of patients with severe or very severe COPD.

Table 2 shows the distribution of the PRO scores obtained at baseline. Almost all scores were shifted toward the milder end of each scale. The best possible score (“floor effect”) was observed except on the SGRQ Total Score and the Hyland Scale, although the best possible score is the ceiling for the BDI. Most of the scores obtained from the PRO measuring tools deteriorated due to the severity of airflow limitation, with the exception of the D-12 Affective Score and E-RS Cough and Sputum.

### 3.2. Episodes Identified during Follow-Up Periods

Of the 122 enrolled patients, 18 (14.8%) were confirmed to have died during the follow-up period. The observed period for mortality was 43.0 ± 44.5 months, with a median of 21.6 months and a range of 4 to 74 months (1324.2 ± 1377.0 days with a range of 138 to 2281 days). An episode of exacerbation was identified in 66 of 117 available subjects (56.4%). The mean duration from entry to last attendance or the first episode of exacerbation was 21.5 ± 16.0 months, ranging from 0 to 74 months (669.3 ± 497.0 days with a range of 7 to 2273 days). Forty-one out of 119 available subjects (34.5%) were hospitalized for exacerbation at least once during the observation period with a mean of 30.5 ± 25.0 months ranging from 1 to 74 months (941.0 ± 761.0 days with a range of 54 to 2273 days).

### 3.3. Predictive Properties of Mortality

Table 3 shows the results of the univariate Cox proportional hazards model in analyzing the association of major clinical measures and scores obtained from PRO measures with mortality. Crude Cox regression analysis of the raw predictors revealed that HR was statistically significant for age, some of the physiological measures, including FVC, FEV_1_, FEV_1_/FVC, and DLco, and scores from some PRO measures such as the BDI, SGRQ Total and Activity, CAT, and Hyland Scale. This demonstrates that these PRO measures are all significant mortality predictors in stable COPD. In other words, health status, global quality of life scale, and some measurements of dyspnea are related to mortality.

It is advisable to strive for standardization of Cox regression analysis using actual measurements to compare variables with different units rather than crude Cox regression analysis of the raw predictors. HRs per SD are shown in Table 3, using the z-score transformation as a general standardization method (Table 3). On the other hand, C-index is often preferred to compare different event prediction models. Among the significant mortality predictors, the C-index for FEV_1_ was the highest at 0.733.

**Figure 1:**
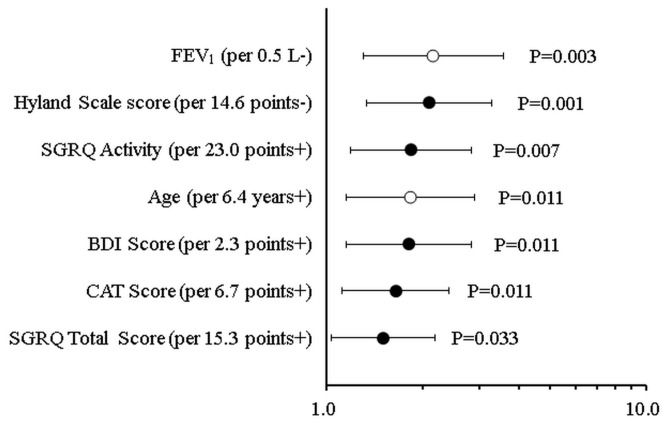
Comparison of the HR (hazard ratio) of mortality associated with significant scores from patient-reported outcome tools and established predictors of mortality such as age as well as FEV_1_.

The comparison of the HR of mortality associated with significant PRO scores and established predictors of mortality, such as age and FEV_1_, is shown in Figure 1. How much risk increases for a 1 SD increase (+) or decrease (−) is indicated in the order of HR per SD change, that is, in the order of the largest change in mortality risk. This can be called a standardized illustration of the magnitude of the effect on mortality.

It is also known that the results of multivariate analysis based on the Cox proportional hazards model are unstable when there are fewer than 20 events, and since there were 18 deaths in the present study, fewer than 20, multivariate analysis was not performed.

### 3.4. Predictive Properties of AECOPD

HRs for much of the clinical information and physiological measures were statistically significant, revealing that the older the patient and the poorer their physiological measures, the greater the risk (Table 4). PaO_2_ and BMI, however, were not associated with a greater risk.

**Figure 2:**
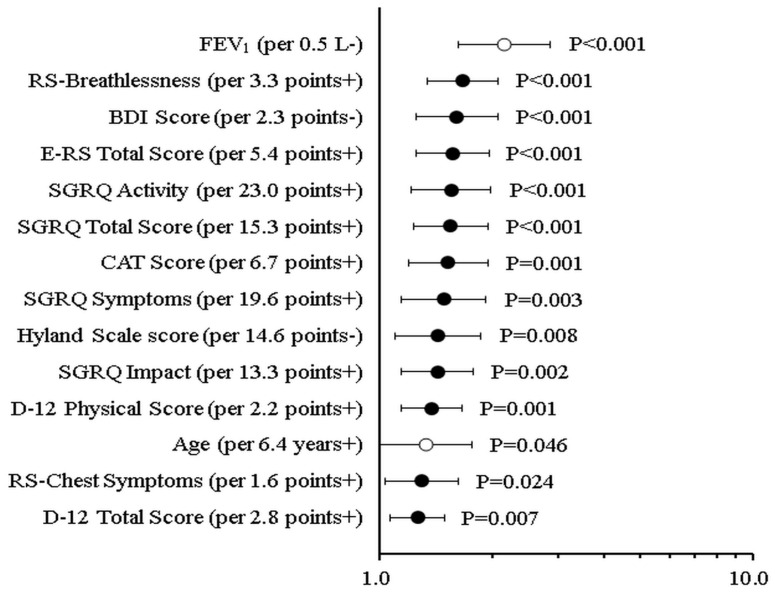
Comparison of the HR (hazard ratio) of the first acute exacerbation of COPD associated with significant scores from patient-reported outcome tools and established predictors of exacerbation such as age as well as FEV_1_.

The majority of PRO tool scores were significantly associated with the first exacerbation, apart from the D-12 Affective score and RS-Cough and Sputum, which did not demonstrate a statistically significant predictive relationship. The highest C-index was 0.754 for FEV_1_. As previously described, Cox regression of standardized predictors with exacerbation is shown in Table 4, and the comparison of the HR of exacerbation associated with significant scores of PROs, age and FEV_1_ is illustrated in Figure 2.

### 3.5. Predictive Properties of the First Hospitalization Due to Acute Exacerbation

**Figure 3:**
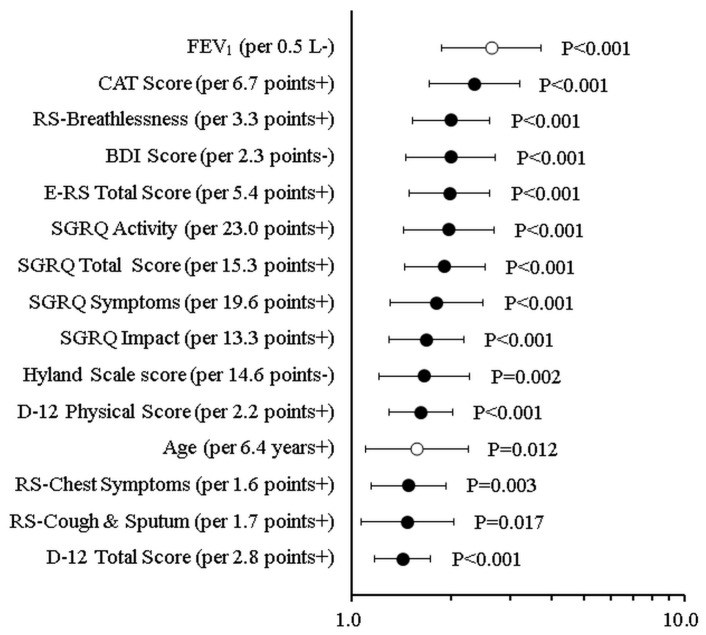
Comparison of the HR (hazard ratio) of the first hospitalization due to acute exacerbation of COPD associated with significant scores from patient-reported outcome tools and established predictors of the first hospitalization for exacerbation such as age as well as FEV_1_.

Statistically significant HRs were observed for all measures, apart from BMI and the D-12 Affective score, in relation to the first hospitalization caused by acute exacerbation. Table 5 and Figure 3 show the results of the univariate analysis based on the Cox proportional hazards model for the data obtained at baseline and the time to hospitalization for the first AECOPD. Almost all clinical, physiological, and PRO measurements obtained at baseline except for BMI and the D-12 Affective score were significant predictors of first hospitalization for exacerbation.

## 4. Discussion

The purpose of the current study was to determine whether PRO measures have risk-predictive ability. Six different PRO measures were examined for 14 scores, including subscales. For mortality, the SGRQ Total and CAT scores assess health status; the BDI score assesses dyspnea; the SGRQ Activity score assesses activity, one of the three components of health status; and the Hyland Scale score, a global score that is considered a very comprehensive assessment of health-related quality of life, were statistically significant predictors. Contrasting these five scores that were concluded to be significantly associated with mortality with the other nine scores for which no significant association could be found, it is necessary to consider the conceptual framework within which each of the scales was designed. The SGRQ Total, CAT scores, and the Hyland Scale score are considered to be a comprehensive overview of both health-related quality of life and health status. It is hypothesized that the importance of these prognostic factors is derived from the fact that these scores encapsulate essential information in a condensed form.

The BDI score, a measure of dyspnea, was a significant predictor of mortality in the current study. It has been suggested that the SGRQ Activity score is analogous to the activity of daily life and can be used to evaluate dyspnea [31]. Reports studying COPD-specific health status or health-related quality of life components have indicated that dyspnea is involved in 30–40% of scores [32]. Given the three positive scores, which are considered comprehensive representations of health status or health-related quality of life, this may reflect the assumption that dyspnea is a significant prognostic factor. 

In contrast, the D-12 Total score and two of its subscales, which assess dyspnea, as well as the RS-Breathlessness score, a subscale of E-RS, were not shown to be significant predictors. Although it is not easy to measure the perception of breathlessness due to its sensory quality and affective components, it has been theorized that the D-12 attempts to scale breathlessness based on descriptions and to be a precise characterization of the sensory and affective dimensions of dyspnea. It has been reported that the BDI score was strongly significantly correlated with mortality, whereas the peak Borg score at the end of progressive cycle ergometry was not [33]. Thus, studies differ in their findings as to whether dyspnea is a statistically significant prognostic factor. When comparing patient-reported outcome tools, we must avoid simple summaries because the results depend not only on the underlying conceptual constructs but also on the measuring properties. In the present study, D-12 showed a highly skewed distribution of scores, which may have led to negative results. Nevertheless, the disparity in the forecasting ability of mortality between tools could have been the result of measuring properties.

The highest C-index for mortality predictors was FEV_1_, suggesting it is a better predictor of mortality than any of the PRO measures studied. Several factors have been reported to be better predictors of mortality than FEV_1_. In 2011, Waschki and colleagues discovered that physical activity is a better predictor of mortality than FEV_1_ and is the best predictor of all-cause mortality [16]. The C-index of FEV_1_ was 0.75 in their study, which is comparable to our own finding of 0.733. Furthermore, they reported that the C-index for both the SGRQ Total and Activity scores was 0.64 and 0.67, respectively, which corresponds to the findings of the present investigation and demonstrates their importance as predictors of mortality. 

The PRO tool with the best predictive ability for mortality considered in this analysis was the Hyland Scale score. The C-index of the FEV_1_ and Hyland Scale scores showed that the former was higher than the latter. In some of the uncorrected measured HRs, Hyland Scale scores may have appeared to be more strongly associated with mortality, especially when examining *p* values. In other words, the prognostic ability of the two is considered to be very similar. In the previous literature, subtle differences in prognostic significance have been reported depending on the population and statistical methodology [16], and the present analysis does not necessarily place a lower value on the predictive value of PRO measures compared to FEV_1_. Therefore, it is not easy to discuss the relative merits of different indices in terms of risk-predictive ability. As some of the relevant indicators change, the corresponding assumptions about how much the prognosis will change must be compared, and it should always be debated what analysis and what assumptions are best, sometimes giving the impression that convenient methods of analysis are chosen according to the preferences of researchers. Several PRO measures were examined for their relationship to mortality, with positive scores on comprehensive measures of health status or health-related quality of life and scores related to dyspnea, while other PRO measures were negative. This negates the short-sighted notion that every PRO measure is predictive, and the constructs of each of the PRO tools will have to be fully considered.

This study also sought to determine the markers of clinically significant exacerbation and hospitalization [34,35,36]. We found that most of the clinical, physiological, and PRO measurements taken at the start were significant predictors of the first exacerbation and the first hospitalization caused by it, apart from BMI and the D-12 Affective score. PaO_2_ and RS-Cough and Sputum were significant hospitalization predictors but not exacerbation predictors. Some reports have indicated an association between specific indicators and the emergence of exacerbation, but, to our knowledge, there have been only a few studies to compare indicators at baseline as an exacerbation predictor. The abundance of risk predictors in a real-world clinical setting may be one of the important results of the present study. In other words, for COPD patients with lower performance on these measures, caution should be exercised as exacerbations are more likely to develop. 

FEV_1_ was found to have the highest C-index in both analyses for predicting exacerbation and hospitalization. This analysis of various PRO measures concluded that the most commonly used FEV_1_ is superior for predicting the risk of AECOPD. The CAT is the PRO measure that is most frequently analyzed as a potential predictor of exacerbation. It has been reported to be capable of predicting exacerbation and hospitalization [37,38,39,40]. However, since the present study demonstrated that many PRO measurement tools could also predict exacerbation, care should be taken not to overemphasize the benefits of the CAT. 

Some limitations of the present study should be mentioned. First, this single-center study was limited by the number of patients with COPD admitted to the study site. Although this is a potential weak point, it contains all patients with stable COPD seen in this hospital during the study period. It is possible, given the small sample size, that there was insufficient power to evaluate any association. Furthermore, because our study included predominantly men, generalizations of these results to women with COPD may be unwarranted. Since the numbers of women with COPD were, in fact, quite low in Japan, the study reflected the reality of clinical COPD in our population. Lastly, although the Hyland Scale score, that is, one of the global quality of life scales, topped the list of risk predictive ability for PRO tools, to the best of our knowledge, there have been no previous reports on the clinical importance of the global quality of life scale for COPD. Because it is so simple, we have used it routinely in our laboratory for many years. Its role in the medical care of patients with COPD is an important topic for further study.

## 5. Conclusions

For mortality, the SGRQ Total and CAT scores assess health status; the BDI score assesses dyspnea; the SGRQ Activity score assesses activity, one of the three components of health status; and the Hyland Scale score, a global score that is considered a very comprehensive assessment of the quality of life related to health, were statistically significant predictors. The Hyland Scale score had the highest risk predictive ability, but the C index did not reach the level of the most commonly used FEV_1_. Almost all clinical, physiological, and PRO measurements obtained at baseline were significant predictors of the first exacerbation and the first hospitalization for exacerbation, with a few exceptions. The results may depend not only on the underlying conceptual constructs but also on the measuring properties.

## Figures and Tables

**Table 1:** Patient characteristics in 122 subjects with COPD at baseline.

		Mean	SD	Max.	Min.
Age	years	74.5	6.4	88.0	58.0
BMI	kg/m^2^	22.5	3.1	31.6	14.0
Cumulative Smoking	pack-years	57.9	29.2	204.0	10.0
FVC	Liters	3.08	0.76	5.34	1.35
FEV_1_	Liters	1.72	0.54	3.13	0.52
FEV_1_/FVC	%	55.8	11.1	69.9	22.4
RV/TLC ^1^	%	46.0	10.0	85.1	18.1
DLco ^2^	mL/min/mmHg	12.47	5.54	37.32	1.94
PaO_2_^3^	mmHg	78.7	8.6	101.5	56.6

^1^ *n* = 121, ^2^ *n* = 120, ^3^ one patient receiving oxygen.

**Table 2:** Score distribution of questionnaires and comparison of scores obtained from patient-reported outcome measurements between GOLD 1, 2 and 3 + 4 groups classified by the severity of airflow limitation.

Patient-Reported Outcomes	Items	Possible Score Range	Score Distribution				GOLD 1 (*N* = 41)		GOLD 2 (*N* = 60)		GOLD 3 + 4 (*N* = 21)	
	(*n*)		Mean	SD	Max.	Min.	Mean	SD	Mean	SD	Mean	SD
D-12 Total Score	12	0–36	1.6	2.8	15.0	0.0	0.9 ± 2.1		1.3 ± 2.2 ^§^		3.9 ± 4.2 ^¶¶^	
D-12 Physical Score	7	0–21	1.4	2.2	10.0	0.0	0.7 ± 1.4		1.2 ± 1.7 ^§^		3.4 ± 3.2 ^¶¶^	
D-12 Affective Score	5	0–15	0.2	0.9	5.0	0.0	0.1 ± 0.8		0.2 ± 0.7		0.5 ± 1.5	
BDI Score	3	0–12	9.3	2.3	12.0	4.0	10.2 ± 2.0		9.4 ± 2.3 ^§^		7.5 ± 2.2 ^¶¶^	
E-RS Total Score ^1^	11	0–40	5.5	5.4	24.0	0.0	3.5 ± 3.5		5.1 ± 5.0 ^§3^		10.3 ± 6.5 ^¶¶^	
E-RS Breathlessness	5	0–17	2.4	3.3	15.0	0.0	1.0 ± 1.7		2.1 ± 2.8 ^§§5^		6.1 ± 4.1 ^¶¶^	
E-RS Cough and Sputum ^2^	3	0–11	2.0	1.7	7.0	0.0	1.7 ± 1.6		2.1 ± 1.8 ^4^		2.1 ± 1.9	
E-RS Chest Symptoms	3	0–12	1.0	1.6	6.0	0.0	0.8 ± 1.4		0.8 ± 1.6 ^§5^		2.0 ± 2.0 ^¶^	
SGRQ Total Score	50	0–100	23.0	15.3	63.1	1.2	16.5 ± 10.4		21.8 ± 14.6 ^§§^		39.1 ± 14.7 ^¶¶^	
SGRQ Symptoms	8	0–100	39.2	19.6	81.4	0.0	33.3 ± 17.0		37.1 ± 18.7 ^§§^		56.4 ± 17.9 ^¶¶^	
SGRQ Activity	16	0–100	31.0	23.0	87.2	0.0	21.3 ± 19.1		29.4 ± 21.6 ^§§^		54.7 ± 17.3 ^¶¶^	
SGRQ Impact	26	0–100	13.6	13.3	55.2	0.0	8.8 ± 8.4		13.0 ± 12.9 ^§^		24.8 ± 16.0 ^¶¶^	
CAT Score	8	0–40	9.0	6.7	27.0	0.0	7.1 ± 5.3		7.9 ± 6.1 ^§§^		15.5 ± 6.9 ^¶¶^	
Hyland Scale Score	1	0–100	67.6	14.6	27.0	30.0	73.4 ± 12.0		68.1 ± 13.6 ^§^		55.0 ± 14.9 ^¶¶^	

GOLD 2 vs. GOLD 3 + 4 (Steel–Dwass test), ^§§^: *p* < 0.001, ^§^: *p* < 0.01 GOLD 1 vs. GOLD 3 + 4 (Steel–Dwass test), ^¶¶^: *p* < 0.001, ^¶^: *p* < 0.05 Kruskal–Wallis test for three groups yields significant differences (*p* < 0.001) except for D-12 Affective Score and E-RS Cough and Sputum. ^1^ *n* = 119, ^2^ *n* = 120, ^3^ *n* = 57, ^4^ *n* = 58, ^5^ *n* = 59.

**Table 3:** Univariate Cox proportional hazards analyses on the relationship between baseline measurements and mortality.

	Crude Cox Regression of the Raw Predictors			Cox Regression of the Standardized Predictors	
	Hazard Ratio (95% CI)	*p* Value	C-Index	SD	Hazard Ratio (95% CI)
Age (years)	1.099 (1.022–1.183)	0.011	0.645	6.4	1.824 (1.146–2.904)
BMI (kg/m^2^)	0.990 (0.843–1.162)	0.901	0.562	3.1	0.969 (0.591–1.589)
Cumulative Smoking (pack-years)	0.998 (0.984–1.012)	0.801	0.498	29.2	0.949 (0.632–1.425)
FVC (Liters)	0.495 (0.251–0.977)	0.043	0.619	0.76	0.585 (0.348–0.982)
FEV_1_ (Liters)	0.244 (0.096–0.615)	0.003	0.733	0.54	0.464 (0.280–0.768)
FEV_1_/FVC (%)	0.955 (0.920–0.992)	0.017	0.726	11.1	0.602 (0.397–0.913)
RV/TLC (%) ^1^	1.015 (0.979–1.054)	0.416	0.610	10.0	1.166 (0.806–1.686)
DLco (mL/min/mmHg) ^2^	0.863 (0.782–0.952)	0.003	0.672	5.54	0.442 (0.256–0.764)
PaO_2_ (mmHg) ^3^	0.983 (0.931–1.038)	0.537	0.571	8.6	0.863 (0.542–1.376)
D-12 Total Score	1.011 (0.893–1.145)	0.859	0.656	2.8	1.032 (0.729–1.462)
D-12 Physical Score	1.070 (0.920–1.245)	0.380	0.661	2.2	1.158 (0.835–1.606)
D-12 Affective Score	0.435 (0.063–2.995)	0.397	0.550	0.9	0.472 (0.083–2.690)
BDI Score	0.777 (0.640–0.943)	0.011	0.690	2.3	0.554 (0.352–0.872)
E-RS Total Score	1.037 (0.972–1.108)	0.272	0.611	5.4	1.219 (0.856–1.735)
RS-Breathlessness	1.079 (0.974–1.195)	0.147	0.603	3.3	1.281 (0.916–1.789)
RS-Cough and Sputum	1.028 (0.791–1.336)	0.837	0.528	1.7	1.048 (0.669–1.642)
RS-Chest Symptoms	1.106 (0.872–1.404)	0.405	0.583	1.6	1.179 (0.800–1.738)
SGRQ Total Score	1.027 (1.002–1.052)	0.033	0.683	15.3	1.499 (1.033–2.177)
SGRQ Symptoms	1.024 (1.000–1.048)	0.053	0.669	19.6	1.579 (0.994–2.507)
SGRQ Activity	1.027 (1.007–1.046)	0.007	0.706	23.0	1.832 (1.184–2.835)
SGRQ Impact	1.016 (0.988–1.044)	0.274	0.621	13.3	1.227 (0.850–1.771)
CAT Score	1.077 (1.017–1.142)	0.011	0.699	6.7	1.643 (1.118–2.418)
Hyland Scale Score	0.951 (0.922–0.980)	0.001	0.694	14.6	0.477 (0.304–0.750)

^1^ *n* = 121, ^2^ *n* = 120, ^3^ one patient receiving oxygen.

**Table 4:** Univariate Cox proportional hazards analyses on the relationship between baseline measurements and acute exacerbation of COPD.

	Crude Cox Regression of the Raw Predictors			Cox Regression of the Standardized Predictors	
	Hazard Ratio (95% CI)	*p* Value	C-Index	SD	Hazard Ratio (95% CI)
Age (years)	1.046 (1.001–1.093)	0.046	0.578	6.4	1.331 (1.005–1.761)
BMI (kg/m^2^)	0.936 (0.865–1.014)	0.104	0.580	3.1	0.816 (0.638–1.043)
Cumulative Smoking (pack-years)	0.996 (0.987–1.005)	0.357	0.534	29.2	0.887 (0.689–1.144)
FVC (Liters)	0.500 (0.344–0.725)	<0.001	0.673	0.76	0.589 (0.444–0.783)
FEV_1_ (Liters)	0.242 (0.144–0.410)	<0.001	0.717	0.54	0.463 (0.348–0.615)
FEV_1_/FVC (%)	0.951 (0.930–0.972)	<0.001	0.661	11.1	0.573 (0.448–0.732)
RV/TLC (%) ^1^	1.039 (1.015–1.063)	0.001	0.641	10.0	1.462 (1.160–1.844)
DLco (mL/min/mmHg) ^2^	0.924 (0.875–0.977)	0.005	0.606	5.54	0.647 (0.478–0.877)
PaO_2_ (mmHg) ^3^	0.976 (0.949–1.004)	0.090	0.573	8.6	0.812 (0.638–1.033)
D-12 Total Score	1.087 (1.023–1.154)	0.007	0.633	2.8	1.263 (1.066–1.496)
D-12 Physical Score	1.160 (1.064–1.265)	0.001	0.636	2.2	1.379 (1.143–1.663)
D-12 Affective Score	1.105 (0.909–1.345)	0.316	0.515	0.9	1.095 (0.917–1.306)
BDI Score	0.816 (0.732–0.909)	<0.001	0.664	2.3	0.621 (0.482–0.800)
E-RS Total Score	1.087 (1.043–1.133)	<0.001	0.638	5.4	1.571 (1.257–1.964)
RS-Breathlessness	1.169 (1.094–1.250)	<0.001	0.643	3.3	1.668 (1.343–2.073)
RS-Cough and Sputum	1.151 (0.997–1.329)	0.054	0.573	1.7	1.273 (0.996–1.627)
RS-Chest Symptoms	1.172 (1.021–1.344)	0.024	0.561	1.6	1.294 (1.035–1.620)
SGRQ Total Score	1.029 (1.014–1.045)	<0.001	0.660	15.3	1.551(1.230–1.954)
SGRQ Symptoms	1.020 (1.007–1.034)	0.003	0.630	19.6	1.483 (1.142–1.926)
SGRQ Activity	1.019 (1.009–1.030)	<0.001	0.671	23.0	1.555 (1.219–1.985)
SGRQ Impact	1.027 (1.010–1.045)	0.002	0.609	13.3	1.427(1.140–1.787)
CAT Score	1.066 (1.027–1.106)	0.001	0.624	6.7	1.525 (1.191–1.163)
Hyland Scale Score	0.976 (0.958–0.994)	0.008	0.611	14.6	0.698 (0.535–0.910)

^1^ *n* = 121, ^2^ *n* = 120, ^3^ one patient receiving oxygen.

**Table 5:** Univariate Cox proportional hazards analyses on the relationship between baseline measurements and hospitalization due to acute exacerbation of COPD.

	Crude Cox Regression of the Raw Predictors			Cox Regression of the Standardized Predictors	
	Hazard Ratio (95% CI)	*p* Value	C-Index	SD	Hazard Ratio (95% CI)
Age (years)	1.074 (1.016–1.135)	0.012	0.602	6.4	1.573 (1.105–2.238)
BMI (kg/m^2^)	0.930 (0.834–1.037)	0.191	0.561	3.1	0.799 (0.571–1.119)
Cumulative Smoking (pack-years)	0.993 (0.983–1.004)	0.234	0.532	29.2	0.823 (0.598–1.134)
FVC (Liters)	0.391 (0.248–0.618)	<0.001	0.708	0.76	0.489 (0.345–0.693)
FEV_1_ (Liters)	0.168 (0.090–0.315)	<0.001	0.754	0.54	0.379 (0.269–0.534)
FEV_1_/FVC (%)	0.939 (0.915–0.964)	<0.001	0.690	11.1	0.496 (0.371–0.661)
RV/TLC (%) ^1^	1.055 (1.027–1.085)	<0.001	0.714	10.0	1.710 (1.300–2.249)
DLco (mL/min/mmHg) ^2^	0.885 (0.824–0.951)	0.001	0.638	5.54	0.509 (0.342–0.756)
PaO_2_ (mmHg) ^3^	0.950 (0.916–0.985)	0.005	0.603	8.6	0.643 (0.471–0.876)
D-12 Total Score	1.135 (1.059–1.216)	<0.001	0.698	2.8	1.426 (1.175–1.732)
D–12 Physical Score	1.248 (1.128–1.381)	<0.001	0.700	2.2	1.616 (1.298–2.012)
D–12 Affective Score	1.200 (0.956–1.506)	0.115	0.532	0.9	1.179 (0.961–1.447)
BDI Score	0.745 (0.652–0.851)	<0.001	0.699	2.3	0.503 (0.369–0.685)
E-RS Total Score	1.135 (1.078–1.195)	<0.001	0.701	5.4	1.977 (1.497–2.611)
RS–Breathlessness	1.235 (1.139–1.34)	<0.001	0.692	3.3	1.996 (1.532–2.601)
RS–Cough and Sputum	1.255 (1.042–1.513)	0.017	0.623	1.7	1.475 (1.072–2.030)
RS–Chest Symptoms	1.276 (1.088–1.496)	0.003	0.646	1.6	1.487 (1.147–1.929)
SGRQ Total Score	1.043 (1.024–1.062)	<0.001	0.702	15.3	1.909 (1.445–2.523)
SGRQ Symptoms	1.031 (1.014–1.048)	<0.001	0.684	19.6	1.804 (1.306–2.492)
SGRQ Activity	1.030 (1.016–1.044)	<0.001	0.712	23.0	1.966 (1.439–2.687)
SGRQ Impact	1.040 (1.020–1.060)	<0.001	0.653	13.3	1.677 (1.295–2.172)
CAT Score	1.137 (1.085–1.191)	<0.001	0.712	6.7	2.347 (1.721–3.202)
Hyland Scale Score	0.966 (0.946–0.987)	0.002	0.625	14.6	0.604 (0.442–0.826)

^1^ *n* = 121, ^2^ *n* = 120, ^3^ one patient receiving oxygen.

## Data Availability

Anonymized participant data will be available upon reasonable request to the corresponding author.
